# Dexamethasone restrains ongoing expression of interleukin-23p19 in peripheral blood-derived human macrophages

**DOI:** 10.1186/1471-2210-11-8

**Published:** 2011-07-26

**Authors:** Linda Palma, Carla Sfara, Antonella Antonelli, Mauro Magnani

**Affiliations:** 1 Dipartimento di Scienze Biomolecolari, Università degli Studi di Urbino "Carlo Bo", Via A. Saffi, 2, 61029 Urbino, PU, Italy

## Abstract

**Background:**

Since its recent discovery, interleukin-23 has been shown to be involved in the pathogenesis of autoimmune diseases favoring the development of a T cell subset referred to as T helper 17. Glucocorticoids are widely employed in inflammatory and autoimmune diseases as they inhibit pro-inflammatory signaling and prevent production of inflammation mediators. Very limited information is available about the efficacy of synthetic glucocorticoids in containing the expression of interleukin-23 under cell activation.

**Results:**

We demonstrate here that the glucocorticoid analogue dexamethasone administered to human monocyte-derived macrophages is indeed able to restrain the expression of interleukin-23 once it has been triggered by a pro-inflammatory stimulus. This effect of dexamethasone is here demonstrated being secondary to suppression of p38 MAPK activity, and involving a protein phosphatase - likely MAPK phosphatase-1 (MKP-1).

**Conclusions:**

Results reported in this paper show that a 10 nanomolar dose of dexamethasone not only prevents inflammatory activation but is also efficacious in confining active inflammation. This effect is here demonstrated not to occur through "canonical" inhibition of the NF-κB transcription factor but through a distinct cascade of down-modulation, that underlines the importance of the transactivating activity of glucocorticoid receptor in the context of its anti-inflammatory action.

## Background

IL-23 is a heterodimeric cytokine composed of the IL-12p40 subunit and the recently discovered IL-23p19 subunit, related to IL-12p35 [1]. The IL-23 receptor complex is present on the surface of APC, NK and activated T cells. Despite IL-23p19 expression in different tissues and cell types, p19 alone has not been found to have biological activity and appears to be functional only when dimerized with p40, which occurs in activated Mφ and DC. Production of IL-23 by APC is triggered by host immune stimuli as interferons and by bacterial products, such as LPS, that signal through TLR [2-4]. Whereas IL-12 drives the differentiation of T helper 1 cells (Th1), IL-23 favors the development of a T cells subset with a unique expression profile, characterized by prominent production of IL-17, therefore referred to as Th17 cells [5,6]. IL-17, in turn, induces pro-inflammatory cytokines that contribute to protective response during infection [7,8]. IL-23 is commonly believed to act on memory CD4^+ ^T cells, enhancing the expansion of committed Th17 and production of IL-17 [1,9], although it was also hypothesized that IL-23 role is not merely limited to a survival factor. In fact, several observations suggest that IL-23 may act earlier during Th17 commitment, promoting their effector function [10]. However, IL-23 is absolutely required for providing Th17 a pathogenic phenotype, because in its absence these cells may have regulatory functions. Nonetheless, the IL-23/IL-17 axis is evolutionarily conserved as it provides a rapid response to catastrophic injuries, caused by certain infective agents (e.g. *K. pneumoniae*, *T. gondii*, *S. aureus *and *B. pertussis*) especially in gut, lung and skin.

Although autoimmune diseases have long been considered being T cell-mediated, the pleiotropic role of Mφ in immunity has lately received more attention, and Mφ products have been implicated in various autoimmune pathologies. In particular, it is now demonstrated that Mφ-secreted IL-23, and consequently T cell-derived IL-17, are linked to disease in animal models of autoimmunity [11-13]. Abnormal IL-23 concentration is found also in human biological fluids and tissues affected by rheumatoid arthritis [14], multiple sclerosis [15,16], psoriasis [17], inflammatory bowel diseases [18,19]. Moreover, the IL-23/IL-17 axis plays a role during inflammatory process-derived tumor development: IL-23 decreases number and activity of cytotoxic T cells in transformed tissues [20].

GC hormones modulate a wide array of physiologic functions, especially in the context of immune homeostasis. Therefore, synthetic GC are broadly employed for treatment of inflammatory conditions. Suppression of inflammation by GC is believed to occur through three main mechanisms: non-genomic pathways, direct genomic effects and indirect genomic effects [21]. Ligand-bound GR regulates target genes expression by interaction with GRE. However, genes regulated through this direct interaction are much less (1%) than genes whose expression is indirectly regulated. In fact, the GR is able to influence - mainly inhibit - the activity of several pro-inflammatory transcription factors, including NF-κB, AP-1, t-BET, STAT5 [22,23]: in the promoter environment of pro-inflammatory genes, active GR competes for binding to co-activators, affects RNA polymerase II activity, interacts with co-repressors and hystone de-acetylases, specifically modulating the expression of each gene. Besides affecting transcription, GC reduce post-transcriptional stability of particular sets of mRNA, thus dampening the synthesis of inflammatory proteins after the inflammation has been activated [24-26]. The synthetic GC analog DEX has higher affinity for the receptor, higher anti-inflammatory activity and a longer biological half-life than conventional steroids [27]. As for the effects of DEX on IL-23 signaling, it is demonstrated that the GC analogue affects activation of STAT4 induced by IL-23 in PHA/IL-2 T cells [28], and a study by Ma *et al*. shows that DEX inhibits LPS-induced IL-12p40 production in human monocytes [29].

Given the critical role of deregulated IL-23 production in pathogenesis, we investigated *in vitro *the effect that DEX exerts on expression of IL-23 - in particular, of its unique p19 subunit - by human Mφ. Moreover, since NF-κB and p38 MAPK are involved in the regulation of IL-23p19 expression in various cell types of the monocyte/Mφ lineage [30-33], we focused on the mechanism of DEX inhibition on these pathways with respect to down-modulation of IL-23p19.

## Results

### DEX reduces IL-23 levels *in vitro*

**Figure 1:**
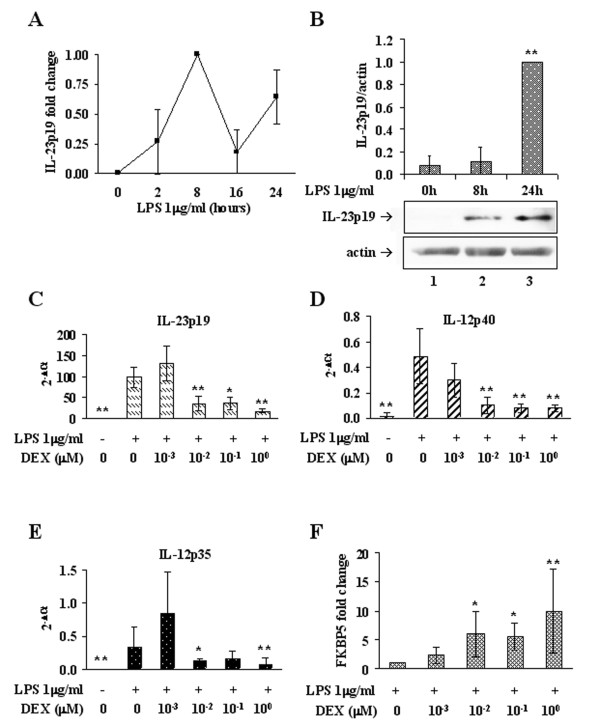
**DEX affects IL-23 mRNA levels**. (A) Time course of IL-23p19 expression in Mφ stimulated with 1 μg/ml LPS for the indicated times. Relative quantification was performed by Real Time RT-PCR according to the ΔΔCt comparative method and data are expressed as fold changes compared to the peak sample (LPS 8h). Data reported in diagram are the average of three independent experiments. (B) Time course of IL-23p19 protein accumulation in Mφ after 24h of stimulation with 1 μg/ml LPS. Western immunoblotting were performed on total cellular extracts. Images are representative of three independent experiments and data are reported in bar graph as fold changes compared to the peak sample (LPS 24h). IL-23p19 signal (upper panel) was normalized to the actin signal (lower panel). (C),(D),(E) DEX-dependent inhibition of expression of the interleukin subunits IL-23p19, IL-12/IL-23p40 and IL-12p35, respectively. Real Time RT-PCR was performed on LPS-stimulated Mφ treated with increasing concentrations of DEX. Results are reported as raw 2^-(Ct _target_-Ct _reference_) to compare the relative abundance of each subunit. Amplification efficiencies of p19 and p35 were comparable, based on the slopes of their standard curves. n = 3. (F) Induction of FKBP5 by DEX in Mφ treated as in (C). Real Time RT-PCR data are reported as fold changes compared to the DEX-untreated sample; n = 6. * p < 0.05, ** p < 0.01.

Primary cultured human Mφ were first subjected to stimulation with LPS in order to assess the time course of IL-23 expression in response to the engagement of TLR-4. Although distinct subjects respond diversely in terms of fold induction, they all show a peak level of IL-23p19 mRNA after 8h of persistent stimulation with LPS (Figure 1A), which is followed by increase of the protein between 8h (lane 2) and 24h (lane 3) (Figure 1B). Therefore, we consider cultured Mφ persistently stimulated with LPS for 8h a suitable *in vitro *model of established IL-23 expression.

The ability of DEX to restrain ongoing IL-23 expression was next evaluated adding the drug during the last 2h of LPS treatment: Mφ stimulated 6h with LPS were either left untreated or treated with 0.001, 0.01, 0.1 or 1 μM DEX, and maintained at 37°C for additional 2h in the presence of LPS. Relative quantification by RT-PCR revealed that, while DEX 0.001 μM does not exert any effect on IL-23p19 expression, DEX 0.01 μM induces a significant decrease in the amount of p19 mRNA compared to DEX-untreated samples (Figure 1C). A similar reduction occurs with 0.1 and 1 μM DEX. On the same samples, the expression of other IL subunits strictly related to IL-23p19 was evaluated and compared by relative quantification, demonstrating that 0.01 μM is the least DEX concentration able to significantly down-regulate IL-12p40 and IL-12p35 mRNA (Figure 1D and 1E). On the other hand, a consistent up-regulation of FKBP5 mRNA was observed with doses ≥ 0.01 μM, demonstrating the effective activation of the GR signaling pathway (Figure 1F).

We then attempted at validating IL-23 expression data also on the protein product, by an IL-23 ELISA on Mφ supernatants collected at 8, 16 and 24h of LPS stimulation, either treated or not with DEX. Unfortunately, the amount of protein was below the limit of sensitivity of the kit, and this problem could not be circumvented by concentrating the samples.

**Figure 2:**
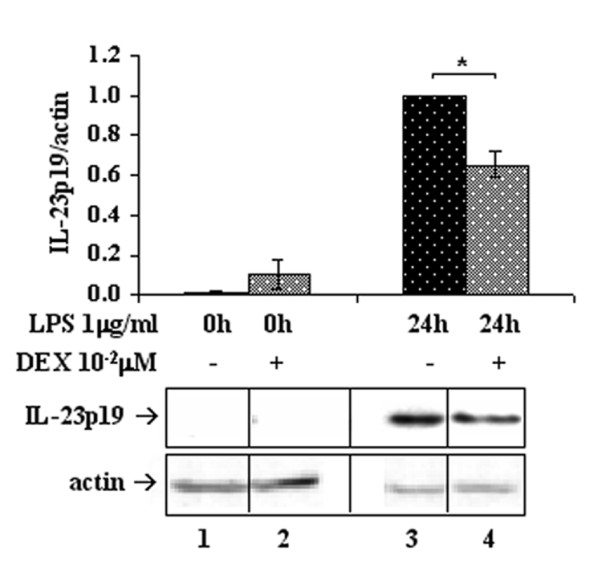
**DEX affects IL-23 protein levels**. DEX (0.01 μM) reduces IL-23p19 protein production. IL-23p19 protein was analyzed by western immunoblotting of Mφ stimulated with LPS (upper panel) for the times indicated. Data obtained from densitometric analyses were normalized on respective actin signals (lower panel). Images are representative of three independent experiments and data are reported in bar graphs as fold changes relative to the peak samples; n = 3. *p < 0.05

However, the effect of DEX on IL-23 protein production was verified at the intracellular level by western immunoblotting of the IL-23p19 subunit. Human Mφ were stimulated 6h with LPS and then either added with 0.01 μM DEX or left DEX-untreated for additional 2h, then all the compounds were removed and total cell lysates were prepared at 24h. As shown in Figure 2, DEX added during IL-23p19 gene transcription leads to a significant decrease of the protein product (lanes 3, 4), confirming the data observed at the transcriptional level and suggesting that the drug can be effective in reducing the amount of secreted interleukin. Interestingly, DEX was also able to prevent IL23-p19 protein accumulation induced in human Mφ by a distinct pro-inflammatory agonist, Zymosan from *Saccharomyces cerevisiae *(data not shown).

### DEX does not affect nuclear localization or DNA-binding of active NF-κB

**Figure 3:**
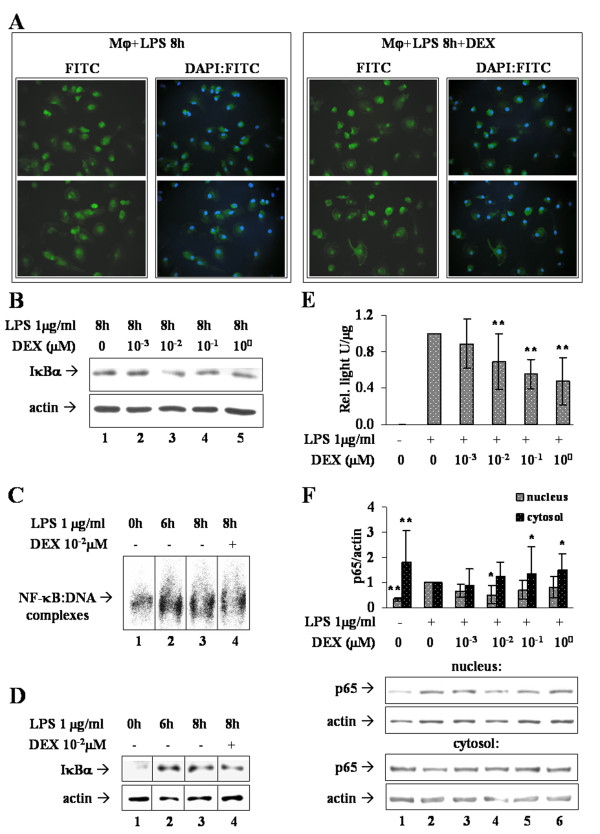
**Mechanism of NF-κB activity restraint by DEX**. (A) Immunofluorescence analyses of NF-κB-p65 sub-cellular localization. FITC pictures show p65 signal in two representative fields of Mφ stimulated 8h with LPS (left box) or of Mφ stimulated 8h with LPS and treated with 0.01 μM DEX (right box). DAPI:FITC pictures were obtained by overlay of the FITC and DAPI signals, and show the nuclear localization of p65. (B). LPS-stimulated Mφ (lane 1) were treated with increasing concentrations of DEX (lanes 2-5) and the amount of IκBα was analyzed by western immunoblotting (upper panel), normalized to the actin signal (lower panel). Image is representative of three independent experiments. (C). Mφ (lane 1, 0h) were subjected to NF-κB EMSA after LPS stimulation for 6h (lane 2) and 8h, in presence (lane 4) or absence (lane 3) of DEX 0.01 μM. The image is representative of four independent sets of samples, each run in triplicate. (D) IκBα western immunoblotting on the same samples employed for EMSA, representative of four independent experiments. (E) NF-κB reporter assay on MM6 cells. NF-κB-driven transcription was triggered by LPS 1 μg/ml for 1h, followed by treatment with indicated DEX concentrations. Data of relative light units/μg of proteins are reported in bar graph as fold changes compared to the DEX-untreated sample (+LPS, 0 DEX); n = 12. (F) NF-κB p65 western immunoblotting on cytosolic and nuclear extracts from the same cells as in (E). Densitometric analysis was followed by normalization on the internal control represented by actin. Data are reported in bar graphs as fold changes relative to the DEX-untreated sample (lane 2); n = 6. * p < 0.05, ** p < 0.01.

Transcription factor NF-κB can be regulated by various events and at distinct levels of the activation cascade. One such mechanism consists of cytoplasmic sequestration through interaction with a member of the IκB family. Immunofluorescence directed to the p65 subunit of NF-κB was performed on Mφ stimulated 6h with LPS and then either added with 0.01 μM DEX or left DEX-untreated for additional 2h. As shown in Figure 3A (left box), nuclear-cytosolic shuttling of p65 is shifted toward nuclei after 8h of LPS treatment. However, addition of DEX does not produce any significant reduction of the nuclear signal (Figure 3A, right box).

Consistent with these results, we observed that none of the tested concentrations of DEX actually up-regulates IκBα, either as mRNA (not shown) or as protein, after 8h of LPS stimulation (Figure 3B), indicating that the lower IL-23p19 expression is not secondary to cytosolic retention of NF-κB. Nonetheless, it could be the consequence of an impaired DNA-binding activity of NF-κB, therefore we performed NF-κB EMSA on Mφ treated as for immunofluorescence (Figure 3C). We observed that the DNA-binding activity of NF-κB before addition of the drug (LPS 6h, lane 2) is up to twice (1.8 ± 0.6, *p *< 0.01, n = 12) that of basal cells (lane 1); when IL-23p19 mRNA is on its peak level (LPS 8h, lane 3) NF-κB activation remains indeed high (1.7 ± 0.4, *p *< 0.05, n = 12), but is not inhibited by DEX (lane 4) (1.7 ± 0.4, *p *< 0.05, n = 12). Surprisingly, the amount of IκBα analyzed on the same cellular extracts (Figure 2D) reflects the transcriptional activity of NF-κB rather than its inhibition. In fact, after 6h of stimulation (lane 2) the amount of IκBα is up to five fold that of basal Mφ (lane 1) (4.6 ± 0.7, *p *< 0.01, n = 4), it remains high at 8h (lane 3) (3.2 ± 2.3, *p *< 0.01, n = 4) and it is almost unaffected by DEX (lane 4) (3.1 ± 1.8, *p *< 0.05, n = 4).

### DEX reduces the transactivation ability of activated NF-κB

To confirm and elucidate the effect of DEX on NF-κB, a reporter cell line was employed. MM6 cells transfected with the LUC coding sequence under NF-κB transcriptional control, were stimulated with LPS over a time course of 8h. At 2h LUC mRNA is at peak level, whereas the maximum of LUC activity is observed at 6-7h (not shown). Since our goal is to evaluate DEX activity on pre-existing inflammatory conditions, we stimulated reporter cells 1h with LPS to trigger LUC expression. Afterward, cells were treated with DEX 0, 0.001, 0.01, 0.1 or 1 μM for one more hour in presence of LPS. Culture medium was then replaced and cells were left at 37°C for additional 5h (Figure 3E). At the end of treatment the luminescent signal is high compared to basal reporter cells, confirming the activation of NF-κB. Whereas DEX 0.001 μM has no effect on the reporter activity, a significant reduction of LUC signal is detectable with DEX ≥ 0.01 μM.

Western immunoblotting for p65 was performed on nuclear and cytosolic extracts from the same cells, to exclude that the lower level of reporter activity was due actually to cytosolic sequestration of NF-κB. As expected, nuclear NF-κB-p65 increases - and the cytosolic decreases - as cells are stimulated with LPS, but the DEX concentrations tested do not modify the sub-cellular localization of p65 to an extent significantly consistent with the reduction of reporter activity (Figure 3F). Therefore, we conclude that DEX reduces the transactivation potential of active NF-κB, because expression of its target gene is down-modulated although the transcription factor remains localized to the nucleus.

### DEX down-modulates p38 MAPK activity

Because p38 MAPK usually promotes NF-κB trans-activity, we speculated that the results above described could arise from p38 impairment by DEX. Thus, in LPS-stimulated Mφ, increasing concentrations of drug were tested on the extent of p38 MAPK phosphorylation, on that of NF-κB p65 phosphorylation on serine residue 276 (Ser276), and on the expression of MAPK phosphatase-1 (MKP-1), a negative regulator of MAPK.

**Figure 4:**
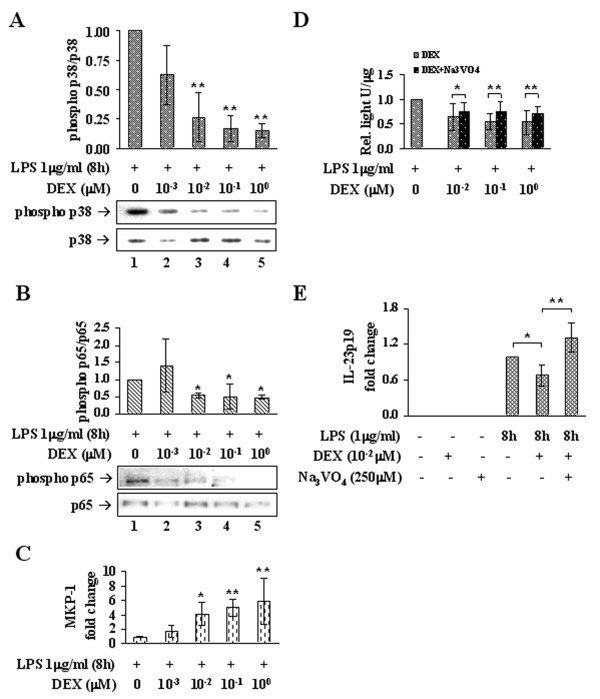
**DEX down-modulates p38 MAPK activity**. (A) DEX-dependent inhibition of p38 MAPK was measured in LPS-stimulated Mφ by western immunoblotting of the phosphorylated protein (phospho Thr180/Tyr182), normalized on the total amount of p38. Data are reported in bar graph as fold changes relative to the DEX-untreated sample (lane 1); n = 3. (B) Inhibition of NF-κB-p65 transactivity by DEX was measured in LPS-stimulated Mφ by western immunoblotting of p65 phosphorylation on Ser276, normalized on the total amount of p65. Data are reported in bar graph as fold changes relative to the DEX-untreated sample (lane 1); n = 3. (C) DEX-induced increase of MKP-1 mRNA determined by RT-PCR on the same samples as in (A). Data are reported as fold changes relative to the DEX-untreated sample (lane 1); n = 6. (D) Bar graph shows data from luciferase assays performed on MM6 reporter cells, stimulated with LPS and treated with increasing concentrations of DEX, administered alone or with sodium orthovanadate. Data are reported as fold changes relative to the DEX-untreated sample (+LPS, 0 DEX); n = 15. (E) Bar graph shows RT-PCR data for the IL-23p19 transcript from Mφ stimulated with LPS and then treated or not with DEX 0.01 μM alone or along with sodium orthovanadate. Data are reported as fold changes relative to the DEX-untreated sample (8 hLPS, - DEX, -Na_3_VO_4_); n = 6. * p < 0.05, ** p < 0.01.

Western blotting was performed on the same type of samples employed for analyses of IL-23 expression. Accordingly, the decreased IL-23 expression is accompanied by a consistent reduction of p38 MAPK phosphorylation. Again, 0.01 μM DEX (Figure 4A, lane 3) promotes a significant dephosphorylation of p38 compared to the control sample (lane 1) and 0.1 and 1 μM DEX (lanes 4, 5) yield similar results. Interestingly, the phosphorylation of p65(Ser276) was decreased with an apparent dose-trend relationship, consistent with the reduction of p38 MAPK activity (Figure 4B).

MKP-1 levels were evaluated by RT-PCR on Mφ under the same experimental conditions as above. As expected, LPS promotes a five-fold increase of MKP-1 mRNA compared to basal Mφ, and DEX administered to the cells during the last 2h of stimulation further enhances MKP-1 expression. In particular, consistent with what above described, at least 0.01 μM DEX is needed to raise significantly the amount of MKP-1 transcript over the level of the DEX-untreated sample, and this induction is maintained by DEX 0.1 and 1 μM (Figure 4C). Furthermore, the involvement of a phosphatase activity in the reduction of NF-κB transactivation potential by DEX was demonstrated on MM6 cells using the phosphatase inhibitor Na_3_VO_4_. MM6 reporter cells were stimulated 1h with LPS and then treated with DEX 0, 0.01, 0.1 or 1 μM, added alone or along with Na_3_VO_4 _for 1h more, in presence of LPS. The medium was then replaced and cells were left at 37°C for additional 5h. Increasing concentrations of DEX promote a significant reduction of the luminescent signal compared to DEX-untreated cells, whereas the phosphatase inhibitor counteracts the trend of such DEX-mediated decrease, as cells treated with Na_3_VO_4 _maintain a higher signal compared to cells treated with DEX alone (Figure 4D).

Similarly, the link between phosphatase induction and IL-23p19 down-modulation was assessed in Mφ, administering Na_3_VO_4 _along with DEX. Mφ were stimulated 6h with LPS and then either left untreated or treated with DEX 0.01 μM in the presence or absence of Na_3_VO_4 _for additional 2h. RT-PCR revealed that DEX produces a decrease of IL-23p19 consistent with that previously observed, compared to DEX-untreated Mφ, whereas addition of the Na_3_VO_4 _along with DEX impedes such decrease, yielding an amount of transcript significantly more abundant not only than that of DEX-treated Mφ but also of control (*p *< 0.05, n = 6) (Figure 4E).

## Discussion

The anti-inflammatory and immunosuppressive properties of GC are attributed to their ability of down-regulating pro-inflammatory pathways, such as those of MAPK, AP-1 and NF-κB, and preventing production and/or release of several pro-inflammatory factors by a variety of cell types challenged with different stimuli [29,34,35].

During the last decade numerous studies have demonstrated that aberrant expression of IL-23 and of its downstream partner IL-17 underlie development and maintenance of autoimmunity. The requirement of IL-23 for Th17 cells to acquire a pathogenic phenotype links unquestionably IL-23 to disease but, to our knowledge, the outcome of DEX treatment on the mechanisms of IL-23 expression is as yet unexplored. Many *in vitro *studies on the effects of DEX perform either a drug treatment -spanning between 30 min to 2h- before activation of inflammatory pathways, or a co-treatment with DEX and pro-inflammatory agonists [29,35-37]. Instead, we reasoned that the performance of DEX could be different if administered after the inflammation has been triggered; therefore, in a therapeutic perspective, we considered more interesting to verify the efficacy of the drug in restraining - rather than preventing - IL-23 expression under active pro-inflammatory signaling. Thus, in our model IL-23p19 expression is stimulated by LPS (Figure 1A and 1B), then the drug is added during the phase of maximal accumulation of the transcript, before its physiological decay. Four logarithms of DEX sub-micromolar concentrations (0.001 to 1 μM) were tested, and the engagement of the GC signaling pathway was verified by the up-regulation of FKBP5, a GR target gene known to be expressed in response to GC as part of the GR auto-regulatory loop [38] (Figure 1F).

We show that at least 0.01 μM DEX is necessary to engage GR (Figure 1F) and to produce a significant reduction of IL-23p19 (Figure 1C). In fact, 0.01-0.1 μM are DEX concentrations within the physiologically and therapeutically relevant range for GR-mediated actions [35,39]. However, the results obtained with DEX 0.01 μM are not improved by higher doses, and this may originate from a saturation effect, hypothesis supported by the evidence that a 0.01 μM plasma concentration of DEX is sufficient to saturate more than 80% of the GR [40].

DEX 0.01 μM reduces the expression of IL-12p40 and IL-12p35 as well (Figure 1D and 1E, respectively). Actually, high level production of functional IL-12 by human monocytes, Mφ and DC requires interferon-γ in addition to microbial stimuli [4,32]. However, this does not exclude that LPS alone may trigger IL-12 expression, though to a lesser extent. In fact, we show that both p40 and p35 are induced by LPS treatment, but p35 is expressed at a level two orders of magnitude lower than p19, based on the respective amplification efficiencies. Unfortunately, since the amplification efficiency of IL-12p40 is not comparable with those of p19 and p35, we cannot make any statement about its relative abundance. However, from these observations we conclude that a physiologic dose of DEX is able to dampen ongoing expression of both IL-23 subunits, through inhibition of the signaling pathways that govern the transcription triggered by microbial stimuli. Moreover, the transcriptional down-modulation of IL-23p19 is here demonstrated to yield a comparable decrease of the protein amount, leading to the conclusion that DEX effectively impairs the ability of producing IL-23 in human Mφ (Figure 2). Therefore, we aimed at identifying the main molecular mechanisms at the basis of IL-23 reduction by DEX.

Consistent with the typical TLR signaling, we have recently shown that NF-κB activity is required for IL-23 expression in LPS-stimulated human Mφ [33]. Thus, we focused on the NF-κB signaling pathway, which DEX inhibits at multiple levels. The transcriptional up-regulation of IκBα has long been known to play a major role in GC-mediated repression of NF-κB, especially in certain cell types [41]. Our research group have demonstrated that in Mφ DEX up-regulates IκBα, which prevents NF-κB triggering by LPS [37]. Instead, when DEX is added over a pre-existing activation of the pathway, down-modulation of pro-inflammatory transcripts is not accompanied by a consistent cytosolic sequestration of NF-κB (Figure 3A). In fact, DEX does not affect amount of IκBα neither at the transcription level nor at that of translation (Figure 3B). Actually, LPS up-regulates IκBα, which is already up to four times more abundant than in basal Mφ when DEX is added to cells (Figure 3D); thus, it is likely that DEX is not able to further enhance IκBα expression. Nonetheless, such high amount of IκBα seems not to be sufficient or competent for NF-κB inhibition in an environment of persistent TLR signaling. In agreement, when NF-κB activation is explored by EMSA (Figure 3C), clearly results that DEX does not impair transcription factor binding to target DNA.

Although the relevance of IκBα up-regulation in GC anti-inflammatory action has been questioned [42,43], our opinion is that it may rather depend on the timing of drug intervention with respect to activation of pro-inflammatory pathways. Accordingly, we verified on a cell-based reporter system that expression of a NF-κB reporter gene is indeed down-modulated upon addition of DEX during LPS stimulation (Figure 3E), in a fashion that does not imply diminished nuclear distribution of the transcription factor (Figure 3F) and hence suggests that NF-κB transactivation ability is decreased.

In synthesis, data from the MM6 cells parallel those from Mφ in indicating that 0.01 μM is the least DEX concentration, among those tested, effective in tethering NF-κB pro-inflammatory activity triggered by LPS. In addition, Mφ data demonstrate that 0.01 μM DEX diminishes IL-23 expression while leaving unaffected NF-κB activity in terms of nuclear localization or DNA-binding; rather, data from the MM6 reporter system suggest that, in an ongoing inflammatory state, DEX acts through reduction of NF-κB trans-activity.

Besides NF-κB, p38 MAPK regulates inflammation through both transcriptional and post-transcriptional mechanisms, and MAPK inhibition has been suggested as a promising anti-inflammatory approach [44,45]. Indeed, p38 MAPK-dependent phosphorylation of proteins in the transactivation complex favors NF-κB activity [46]. For example, MSK1 (mitogen- and stress-activated kinase 1), a downstream target of p38 MAPK, phosphorylates serine 276 of p65 enhancing transcription [36]. In addition, p38 favors inflammation also by post-transcriptional stabilization of pro-inflammatory mRNAs that contain adenylate/uridylate-rich elements in the 3' untranslated region [47-49].

We demonstrate that a decrease of p38 MAPK phosphorylation takes place in LPS-stimulated Mφ upon administration of DEX ≥ 0.01 μM (Figure 4A). Ser276 phosphorylation on NF-κB-p65 normally occurs following activation of p38 MAPK and ERK [50]. As expected, the down-modulation of p38 MAPK is accompanied by a consistent diminution of Ser276 phosphorylation (Figure 4B). Although the role of ERK has not been explored in this work, these results strongly support the involvement of p38 MAPK inhibition in the mechanism of DEX-induced restraint of NF-κB trans-activity and of pro-inflammatory gene expression.

Recent works have focused on the role of MAP kinase phosphatase 1 (MKP-1) in the context of GC anti-inflammatory function. MKP-1 is induced by GC, in fact DEX raises MKP-1 levels in various cell types [51,52]. This phosphatase acts on p38, JNK and ERK, thereby terminating their activation. According to the model here depicted, we show a DEX-dependent up-regulation of MKP-1 (Figure 4C), consistent with the decrease of p38 and p65 phosphorylation. Moreover, addition of Na_3_VO_4_, a phosphatase inhibitor, counteracts the effect of DEX on NF-κB transactivation ability, demonstrating that dephosphorylation of substrates is necessary for the accomplishment of DEX function (Figure 4D). The same mechanism likely applies to the specific expression of endogenous IL-23p19 in Mφ, because the restraint caused by DEX 0.01 μM is completely prevented by Na_3_VO_4 _(Figure 4E).

Because we demonstrated that DEX causes inactivation of p38 MAPK, and p38 favors stability of pro-inflammatory transcripts, we next asked whether the lower amount of IL-23p19 results exclusively from reduced expression - secondary to reduced NF-κB activity - or is actually due to a combination of both impaired synthesis and enhanced degradation. The decay of IL-23p19 mRNA, in the presence or absence of DEX, was analyzed by mRNA decay assays with the inhibitor of gene expression ActD (data not shown). In our experimental approach ActD is added after 30 min from addition of DEX, to allow MKP-1 up-regulation. Unfortunately, due to such particular timing of the assay, we could not conclude whether the rapid (within 30 min) decay in presence of DEX can be ascribed also to enhanced post-transcriptional destabilization of p19 mRNA. A possibility exists that DEX is only capable of preventing stabilization of mRNA-protein complexes, but not of destabilizing pre-existing stable complexes [53]. In light of this, the decreased amount of IL-23p19 may result from failing stabilization of newly synthesized mRNA.

In sum, we show here for the first time that a 0.01 μM concentration of DEX reduces the expression of IL-23 by restraining NF-κB transactivation ability. We demonstrate that NF-κB trans-activity is reduced by loss of Ser276 phosphorylation on p65, and that this is sufficient to switch off the expression of an exogenous NF-κB target gene as well as of the endogenous IL-23p19. We show that this effect requires a phosphatase activity, suggesting that it is mediated by p38 MAPK inhibition, achieved through MKP-1 induction by DEX. From a therapeutic point of view, this latter point is particularly relevant in light of the overexpression of p38α by Mφ of the intestinal lamina propria from inflammatory bowel disease patients [54].

While this paper was in preparation, it was shown that expression of IL12p40 is regulated by a ternary NF-κB complex composed of p65, c-Rel and an hypophosphorylated form of IκBβ that stabilizes the transcription factor [55]. Therefore, it will be intriguing to verify whether IκBβ plays a role in IL-23p19 expression, and eventually to explore the impact of DEX on the phosphorylation status of IκBβ.

## Conclusions

Since continuous priming and recruitment of new T cells into the effectors pool underlies chronic autoimmunity and is involved in the relapsing nature of diseases, gains of interest the rationale of IL-23 neutralization to prevent relapsing-remitting autoimmunities [56]. To this regard, the efficacy of DEX treatment *in vitro *is here demonstrated, with interesting implications from a therapeutic perspective.

Novel GR ligands that selectively promote transrepression but not transactivation have been proposed to maintain anti-inflammatory effects while causing fewer side effects; however, our findings show that DEX-engaged GR induces an anti-inflammatory factor, MKP-1, indeed supporting the opinion that dimerization-deficient GR ligands might not be effective [57], as anti-inflammatory functions of GR are not independent of its dimerization and transactivation activity.

## Methods

### Cell cultures and reagents

Human monocyte-derived Mφ were obtained from healthy blood. Adult volunteers signed an informed consent form before donation at the blood collection centre of Hospital S. Maria della Misericordia of Urbino (Italy), and samples were provided as anonymous. Mφ were prepared by density gradient separation using Lymphoprep solution (specific density, 1.077; Axis-Shield PoC AS, Oslo, Norway). Cells were resuspended in RPMI-1640 medium supplemented with 10% (v/v) heat-inactivated FBS, 100 U/ml penicillin, 100 μg/ml streptomycin and 2 mM L-glutamine. Monocytes were separated by plastic adherence to tissue culture dishes (Sarstedt AG & Co., Nümbrecht, Germany) and/or chamber slides (Nalge Nunc International, Termo Fisher Scientific, Rochester, NY, USA), overnight at 37°C in a humidified 5% CO_2 _atmosphere. Non-adherent cells were removed by repeated washes. Cells were cultured for 7 days, after which > 95% of adherent cells were differentiated Mφ, as revealed by immunostaining and surface marker analyses.

MM6 cells, derived from human acute monocytic leukemia, were obtained from DMSZ GmbH (Braunschweig, Germany) and cultured at a density of 0.5-1 × 10^6 ^cells/ml in a 5% CO_2 _atmosphere at 37°C, in RPMI-1640 supplemented with 10% heat-inactivated FBS, 2 mM L-glutamine, 100 μg/ml streptomycin, 100 U/ml penicillin, 1 × non-essential aminoacids, 9 μg/ml insulin (Sigma-Aldrich, St. Louis, MO, USA), 1 mM sodium pyruvate. All cell culture reagents were from Lonza (Basel, Switzerland).

MM6 cells were transfected with a vector containing the luciferase coding sequence downstream of four tandem repeats of the -κB consensus element. Transfection was performed with Effectene Transfection Reagent (Qiagen GmbH, Hilden, Germany), according to manufacturer's instructions.

Approximately 10^6 monocyte-derived Mφ, or 10^6 MM6 reporter cells, were stimulated with 1 μg/ml of LPS (serotype 0111:B4, Sigma-Aldrich), for the times indicated. DEX (Sigma-Aldrich) was resuspended in PBS to 1 mM and working dilutions were dosed spectrofotometrically at 239 nm. Na_3_VO_4 _(Sigma-Aldrich) was activated according to the standard procedure prior to use at a final concentration of 250 μM.

### Immunofluorescence

The p65 subunit of NF-κB was detected in human Mφ by indirect immunofluorescence. 5 × 10^4 ^cells were washed in PBS, fixed with 4% formaldehyde in PBS for 20 min at room temperature, and permeabilized with 0.5% NP-40 in PBS for 10 min. Samples were blocked with 1% (w/v) BSA and 0.1% (w/v) gelatin in PBS for 1h, then incubated 1h at room temperature with a rabbit polyclonal anti-p65 (Santa Cruz Biotechnology, Santa Cruz, CA, USA), 1:100 in 1% BSA/PBS. Secondary antibody was a FITC-conjugated goat anti-rabbit IgG (Sigma-Aldrich) 1:300 in 1% BSA/PBS, incubated 1h at room temperature. DNA was stained with DAPI (Sigma-Aldrich) at a final concentration 0.2 μg/ml. Samples were fixed in gelvatol and observed with an Olympus IX51 Fluorescence Microscope. FITC and DAPI images were compared by overlay.

### RT-PCR

Total RNA from Mφ was prepared with RNeasy Plus Extraction kit (Qiagen) following the manufacturer's instructions. 0.5 μg of RNA was reverse transcribed in a reaction mix assembled as described in [58]. Real Time PCR analyses were performed in an ABI PRISM 7700 Sequence Detection System 1.9 (Applied Biosystems, Life Technologies, Carlsbad, CA, USA). PCR reactions were assembled in a 25 μl volume using the Hot Rescue Real Time PCR SYBR Green Kit (Diatheva, Fano, Italy) and 0.2 μM of each primer. Primer oligonucleotides were purchased from Sigma-Aldrich and sequences are reported in Table 1. For each sample three replicates were run, corresponding to 10 nanograms of total RNA. Thermal cycling was performed as follows: 10 min at 95°C; 45 cycles of denaturation at 95°C for 15s, annealing for 30s at the temperatures indicated in Table 1 and extension at 72°C for 30s. At the end of PCR cycles, a melting curve was generated to verify the specificity of PCR products. Relative quantification of selected mRNAs was performed according to the ΔΔCt comparative method.

**Table 1:** PCR amplification primers.

Target gene	Primer sequences	T*a* (°C)
*FKBP5*	Fwd 5'-*CCCTCGAATGCAACTCTCTT-*3' Rev 5'-*TCTCCTTTCCGTTTGGTTCT-*3'	62

*GAPDH*	Fwd 5'-*TGCACCACCAACTGCTTAG-*3' Rev 5'-*GATGCAGATGATGATGTTC-*3'	57

*IL-12p35*	Fwd 5'-*CATGCTGGCAGTTATTGA-*3' Rev 5'-*AAGTATGCAGAGCTTGATTTT-*3'	57

*IL-12p40*	Fwd 5'-*AGGAGAGTCTGCCCATTGAGG-*3' Rev 5'-*GACCTCCACCTGCCGAGAA-*3'	65

*IL-23p19*	Fwd 5'-*CGTCTCCTTCTCCGCTTCA-*3' Rev 5'-*GTGCCTGGGGTGGTAGATTT-*3'	65

*MKP-1*	Fwd 5'-*AGCAGAGGCGAAGCATCATC-*3' Rev 5'-*CCCAGCCTCTGCCGAAC-*3'	61

a) Fwd: forward primer.

b) Rev: reverse primer.

c) T*a*: annealing temperature.

d) GAPDH: internal reference gene.

### Assay of IL-23 production

10^6 ^Mφ, plated as above described, were stimulated for 6h with 1 μg/ml LPS, and either treated or not with DEX for two more hours. The culture medium was collected at 8h or replaced and collected at 16 or 24h. IL-23 concentration was measured with the IL-23 Heterodimer Immunoassay kit, according to manufacturer's instructions (Invitrogen Life Technologies, Carlsbad, CA, USA).

### Western immunoblotting and EMSA

Denatured whole cell extracts for western blotting analyses and native whole cell extracts for EMSA were prepared as elsewhere described [58] and protein concentration was determined according to the Lowry [59] and Bradford [60] methods, respectively. Cytosolic and nuclear extracts from MM6 cells were prepared as follows: cells were collected in microfuge tubes and pelleted, washed with ice-cold PBS and incubated 10 min on ice in buffer A (10 mM Hepes/KOH, pH 7.9, 1.5 mM MgCl_2_, 10 mM KCl, 0.5 mM DTT, 0.5 mM PMSF, 0.2 mM EDTA, 10 μg/ml leupeptin, 10 μg/ml pepstatin, 0.1% Nonidet P-40). Lysates were then centrifuged 10 min at 12,000 rpm, 4°C. The cytosolic fraction was removed to fresh tubes whereas pelleted nuclei were resuspended in buffer B (20 mM Hepes/KOH pH 7.9, 25% glycerol, 0.42 M NaCl, 1.5 mM MgCl_2_, 0.5 mM DTT, 0.2 mM EDTA, 0.5 mM PMSF, 10 μg/ml leupeptin, 10 μg/ml pepstatin), incubated 20 min on ice and centrifuged 10 min at 12,000 rpm, 4°C, finally collecting the supernatants (nuclear extracts) in fresh tubes.

Western blotting was performed on cell extracts (15-60 μg, depending on the protein of interest) diluted in 2 × sample buffer, boiled, subjected to 10% SDS-polyacrylamide gel electrophoresis, and electrotransferred to a 0.2 μm nitrocellulose membrane (Bio-Rad, Hercules, CA, USA). The membrane was then blocked with 5% non-fat dry milk or 5% BSA in TBS-0.1% Tween-20 (Sigma-Aldrich). Antibody against IL-23p19 was a mouse monoclonal from BioLegend (San Diego, CA, USA). IκBα (C-21) and p65 (C-20) Ab were rabbit polyclonal from Santa Cruz Biotechnology and for a loading control a rabbit polyclonal anti-actin (Sigma-Aldrich) was utilized. Phosphorylation of p65 was determined with a Phospho-Ser276-specific rabbit polyclonal Ab (#3037), whereas that of p38 MAPK was determined with a Phospho-Thr180/Tyr182-specific mouse monoclonal Ab (#9216) and with a p38 rabbit polyclonal Ab (#9212) (Cell Signaling Technology, Boston, MA, USA). Secondary anti-rabbit and anti-mouse HRP-conjugated Ab were from Bio-Rad and ECL reagents were from GE Healthcare (Little Chalfont, UK). Densitometric analyses were performed with the ChemiDoc System (Bio-Rad).

NF-κB EMSA reactions were assembled as in [58] with 10 μg of native whole cell extracts. DNA-protein complexes were resolved by electrophoresis on 5% non-denaturating polyacrilamide gels, in a TBE buffer system, at a constant voltage of 170 V, for 3 h at 4°C. Gels were then dried and subjected to autoradiography in the GS250 Molecular Imager system, followed by densitometric analysis with the Molecular Analyst software (Bio-Rad).

### LUC assay

LUC assays were performed with the Luciferase Assay System (Promega, Madison, WI, USA) according to the manufacturer's instruction. Briefly, at the end of the treatment cells were collected, washed in ice cold PBS and lysed in 1 × Reporter Buffer. 10 μl of cleared lysate, corresponding to 10-20 μg of proteins, were read in a 96-well plate luminometer (BMG Labtech GmbH, Offenburg, Germany). The luminescent signal was normalized to the total amount of proteins per well, determined according to the Bradford method.

### Statistical analysis

Data are reported as the average ± s.d. of at least three independent experiments and - unless otherwise stated - are expressed as fold changes relative to the calibrator sample each time indicated. The Friedman statistical test was followed by the Dunn's multiple comparison and a *p *value < 0.05 was considered to be significant.

## Abbreviations

**Ab: **antibody; **ActD: **actinomycin D; **APC: **antigen presenting cell; **DC: **dendritic cell; **DEX: **dexamethasone; **GC: **glucocorticoid; **GR: **glucocorticoid receptor; **GRE: **glucocorticoid response element; **IF: **immunofluorescence; **IκB: **inhibitor of -κB; **IL-23: **interleukin-23; **LPS: **lipopolysaccharide; **LUC: **luciferase; **Mφ: **macrophage; **MKP-1: **MAPK phosphatase 1; **MM6: **monomac 6 cells; **Na_3_VO_4_: **sodium orthovanadate; **NF-κB: **nuclear factor -κB; **NK: **natural killer cell; **PBS: **phosphate buffered saline; **TLR: **Toll-like receptor

## Authors' contributions

Design of the study, RT-PCR, western blotting, EMSA, reporter cells were by LP. AA and CS contributed to design of the study and performed macrophage cultures, immunofluorescence and ELISA. Conception, design and coordination were by principal investigator MM. All authors read and approved the final manuscript.
